# Kinetic modelling of phospholipid synthesis in *Plasmodium knowlesi* unravels crucial steps and relative importance of multiple pathways

**DOI:** 10.1186/1752-0509-7-123

**Published:** 2013-11-09

**Authors:** Partho Sen, Henri J Vial, Ovidiu Radulescu

**Affiliations:** 1 Dynamique des Interactions Membranaires Normales et Pathologiques, UMR 5235 CNRS, UM1, UM2, CP 107, Place Eugène Bataillon, 34095 Montpellier Cedex 5, France

**Keywords:** Malaria, Phospholipid metabolism, Plasmodium knowlesi, Mathematical model, Fluxomics, Hybrid optimization

## Abstract

**Background:**

*Plasmodium* is the causal parasite of malaria, infectious disease responsible for the death of up to one million people each year. Glycerophospholipid and consequently membrane biosynthesis are essential for the survival of the parasite and are targeted by a new class of antimalarial drugs developed in our lab. In order to understand the highly redundant phospholipid synthethic pathways and eventual mechanism of resistance to various drugs, an organism specific kinetic model of these metabolic pathways need to be developed in *Plasmodium* species.

**Results:**

Fluxomic data were used to build a quantitative kinetic model of glycerophospholipid pathways in *Plasmodium knowlesi*. *In vitro* incorporation dynamics of phospholipids unravels multiple synthetic pathways. A detailed metabolic network with values of the kinetic parameters (maximum rates and Michaelis constants) has been built. In order to obtain a global search in the parameter space, we have designed a hybrid, discrete and continuous, optimization method. Discrete parameters were used to sample the cone of admissible fluxes, whereas the continuous Michaelis and maximum rates constants were obtained by local minimization of an objective function.The model was used to predict the distribution of fluxes within the network of various metabolic precursors.

The quantitative analysis was used to understand eventual links between different pathways. The major source of phosphatidylcholine (PC) is the CDP-choline Kennedy pathway.

*In silico* knock-out experiments showed comparable importance of phosphoethanolamine-N-methyltransferase (PMT) and phosphatidylethanolamine-N-methyltransferase (PEMT) for PC synthesis.

The flux values indicate that, major part of serine derived phosphatidylethanolamine (PE) is formed via serine decarboxylation, whereas major part of phosphatidylserine (PS) is formed by base-exchange reactions.

Sensitivity analysis of CDP-choline pathway shows that the carrier-mediated choline entry into the parasite and the phosphocholine cytidylyltransferase reaction have the largest sensitivity coefficients in this pathway, but does not distinguish a reaction as an unique rate-limiting step.

**Conclusion:**

We provide a fully parametrized kinetic model for the multiple phospholipid synthetic pathways in *P. knowlesi*. This model has been used to clarify the relative importance of the various reactions in these metabolic pathways. Future work extensions of this modelling strategy will serve to elucidate the regulatory mechanisms governing the development of *Plasmodium* during its blood stages, as well as the mechanisms of action of drugs on membrane biosynthetic pathways and eventual mechanisms of resistance.

## Background

Malaria, caused by protozoan parasites of the genus *Plasmodium*, is one of the most prevalent diseases in the world, with approximately 50% of the world’s population at risk with several cases of malaria worldwide causing a million deaths, mostly children under 5 years old [1]. Four species of *Plasmodium*, *P. falciparum*, *P. vivax*, *P. ovale* and *P. malariae* commonly infect humans, and a fifth, *P. knowlesi*, has recently been identified as being responsible for a significant number of human cases in South-East Asia [2]. There are increasing reports that natural incidence of the primate *P. knowlesi* parasite among humans, can cause severe cases [3].

Malaria parasites have a complex lifecycle involving stages in humans and mosquitoes, with the human blood stage of the infection responsible for much of the disease pathology. During this stage, the parasite develops through the “ring” and “trophozoite” stages, and then divides inside the red blood cells of its host to form an average of ≈ 20 daughter cells in the “schizont” stage. This requires temporally controlled metabolic programs which are co-ordinated, leading to the duplication of the structural components [4]. Transcription and post-transcriptional control of specific mRNAs, as well as translation regulation modulate the gene expression program, such that practically all the genes show some regulation over the course of development [5,6].

The malarial lipid composition has been substantially documented for *P. falciparum* and *P. knowlesi* at their blood stages [7-9].

Glycerophospholipids(PL) are the main *Plasmodium* membrane constituents, with a preponderance of PC and PE and with an increase in phosphatidylinositol (PI) involved in signaling. Upon *P. falciparum* or *P. knowlesi* infection the phospholipid content in the erythrocytes increases to 6-fold. In purified parasites, the main PLs are PC (40–50%), PE (35–45%), PI (4–11%), and SM and PS (< 5*%*). Interestingly, the PE content is unusually high compared with its level in other eukaryotes [10-12]. A major increase in neutral lipids is also detected, but the final amount of these neutral PLs remain very low as compared to the total PLs [13,14].

These lipids mostly originate from the parasite enzymatic machinery, which relies on scavenging and downstream metabolism of polar heads and fatty acids serving as building units throughout a bewildering number of pathways. The PL synthesis machinery in *Plasmodium* is of prime importance since the parasite membranes are almost exclusively composed of PE and PC with quasi-absence of others structural lipids such as cholesterol. Besides, the lipid-derived signaling molecules phosphoinositide exert crucial function regulating parasite development and proliferation that are currently deciphered [15-18].

Phospholipid biogenesis is crucial for the intracellular development of the parasite and constitutes a potential area for therapeutic intervention that has already been validated leading to a novel and promising pharmaceutical approach for the treatment of malaria [19,20].

At the blood stage, *Plasmodium* species thus display a puzzling number of metabolic pathways that are rarely found together in a single organism [21]: (i) the ancestral prokaryotic-type CDP- diacylglycerol dependent pathway; (ii) the eukaryotic-type de novo CDP-choline and CDP-ethanolamine (Kennedy) pathways; (iii) *P. falciparum* and *P. knowlesi* exhibit additional reactions that bridge some of these routes. A plant-like pathway that relies on serine to provide additional PC and PE, is named the serine decarboxylase-phosphoethanolamine methyltransferase (SDPM) pathway. This route is of great interest as it involves serine decarboxylase (SD) that has been characterized in plants and is distributed sporadically throughout animal genomes [21,22]. In addition, base-exchange mechanisms are largely unexplored in *Plasmodium* but are currently explored in our laboratory.

Thus, the malaria parasite possesses a panoply of complex metabolic pathways and at least some of them are of vital importance for the parasite [21]. Based on quantitative biochemical studies available on *P. knowlesi* parasites, we establish here the comprehensive network of metabolic pathways, synthesizing phospholipids at the blood developmental stage [23]. This gives insights on the relative contributions of the different metabolic branches and as to how metabolite levels and metabolic fluxes are modulated and lead to identification of key regulatory steps, resulting in a global view of regulations and metabolic schemes in *Plasmodium*.

## Methods

### Finding parameters and stationary flux profiles from radiolabelled metabolites profiles

In the last decades, isotope labelling became a major tool for studying metabolic activity of many organisms from bacteria to human [24,25]. Such experimental techniques have as primary aim the quantification of intracellular metabolic fluxes. The mathematical analysis of the data can be intricate and depends on the technique (radioactive precursor incorporation, isotopomers distribution) and on the type of experimental measurement (steady state, transient data), therefore there is no unique equation or algorithm allowing to extract information from any fluxomic data. We have based our modelling work on a series of experimental studies designed to elucidate the synthetic pathways of phospholipids in the malaria parasite [8]. The experimental protocol in these studies uses incorporation of PL radiolabelled precursors and measurement of concentration of end products and intermediate labelled metabolites in the metabolic network. Most of the available data consist of concentrations of metabolites after a relatively long incorporation time, for various external concentrations of the precursors.

In order to model the incorporation of precursors we use a kinetic metabolic network model. The time dependent variables of this model are the concentrations of various metabolites inside the parasite. We gather these concentrations in a column vector $c^{\text{in}}\;=\;{\left(c_{1}^{\text{in}},c_{2}^{\text{in}},\ldots ,c_{n}^{\text{in}}\right)}^{T}\;$, where $c_{i}^{\text{in}},\;i\in \;[\;1,n]$ denote concentrations of metabolites *i*. Some metabolites, namely the scavenged precursors, are considered to be kept at constant concentrations outside the parasites. The corresponding concentration vector is $c^{\text{ext}}={\left(c_{1}^{\text{ext}},c_{2}^{\text{ext}},\ldots ,c_{m}^{\text{ext}}\right)}^{T}$, where $c_{i}^{\text{ext}},\;i\;\in \;[\;1,m]$ are the fixed, external concentrations of metabolites *i*. In a typical experiment, concentrations ***c***^*i* *n*^ are measured after a given time, for several values of ***c***^*e* *x* *t*^. The list of metabolites for our PL metabolic network is given in the Table 1. There are three types of external precursors (serine, ethanolamine, and choline), therefore for the study presented in this paper, *m*=3. The model is a network of biochemical reactions. To simplify, all the reactions of the network are modelled as single substrate enzymatic reactions. By doing this, we implicitly assume that cofactors are either not limiting or not time dependent. This constraint could be released in more realistic models, for instance when studying the crosstalk between several pathways. We also consider that all the enzymatic reactions have Henri-Michaelis-Menten kinetics [26,27]:

(1) $$R(c)=\frac{V_{\text{max}}c}{K_{m}+c}$$

**Table 1:** List of reactions and metabolites in the network

**Reaction ID**	**Substrates & products**	**Enzyme (E.C number)**	**ORF (P.falciparum/P.knowlesi)**	**References**
R1	SerE → Ser	?	?	[28]
	(Serine transport)			
R2	Ser → PS	PSS	(MAL8P1.58) /(PKH_051100)	Vial (unpublished data).
	(Phosphatidylserine synthesis)		*predicted*	
R3	Ser → Etn	SD	?	[28]
	(Serine decarboxylation)	4.1.1		
R4	Etn → PEtn	EK	PfEK	[29]
	(Ethanolamine phosphorylation)	2.7.1.82	(PF11_0257) /(PKH_092210)	
R5	PEtn → PCho	PMT	PfPMT	[30,31]
	(Phosphoethanolamine methylation)	2.1.1.103	(MAL13P1.214) / (PKH_121150)	
R6	PS → PE	PSD	PfPSD	[32]
	(Phosphatidylserine decarboxylation)	4.1.1.65	(PFI1370c) /(PKH_072580)	
R7	PEtn → PE	ECT	PfECT	[28,33]
		2.7.7.14	(PF13_0253)/ (PKH_120620)	
		CEPT/EPT	PfCEPT	
		2.7.8.1	(PFF1375c)/(PKH_112100)	
R8	PCho → PC	CCT	PfCCT	[33-35]
		2.7.7.15	(MAL13P1.86)/(PKH_141580)	
		CEPT/CPT	PfCEPT	
		2.7.8.2	(PFF1375c)/(PKH_112100)	
R9	PE → PC	PEMT/PLMT	?	[28,36,37]
	(Methylation)	2.1.1.17/2.1.1.71		
R10	PC → PS	PSSbe	(MAL13P1.335) /(PKH_110380)	
	(base exchange)	2.7.8		
R11	PE → PS	PSSbe	(MAL13P1.335) /(PKH_110380)	
	(base exchange)	2.7.8		Berry and Vial (unpublished data)
R12	PC →	?	?	
	(Phosphatidylcholine efflux)			
R13	PS →	?	?	
	(Phosphatidylserine efflux)			
R14	PE →	?	?	
	(Phosphatidylethanolamine efflux)			
R15	ChoE → Cho	NPP/OCT/CTL	(PFE0825w) /(PKH_101630)	[20,38-40]
	(Choline transport)			
R16	Cho → PCho	CK	PfCK	[29,41]
	(Choline phosphorylation)	2.7.1.32		
R17	EtnE → Etn		?	
	(Ethanolamine diffusion)			

We have used the following abbreviations. SerE = exogenous serine, Ser = intracellular serine, PS = phosphatidylserine, EtnE = exogenous ethanolamine, Etn = intracellullar ethanolamine, PEtn = phosphoethanolamine, PE = phosphatidylethanolamine, ChoE = exogenous choline, Cho = intracellular choline, PCho = phosphocholine, PC = phosphatidylcholine, DAG = diacylglycerol, SD = serine decarboxylase, PSSbe = phosphatidylserine synthase I, PMT = phosphoethanolamine-N-methyltransferase, PEMT = phosphophatidylethanolamine-N-methyltransferase, CCT = cholinephosphate cytidylyl transferase, ECT = ethanolaminephosphate cytidylyl transferase, CEPT = choline/ethanolamine phosphotransferase, CK = choline kinase, EK = ethanolamine kinase, NPP = New permeation pathway, OCT = Organic cationic transporter, ? = putative genes found.

This model was deposited in BioModels Database [42] and assigned the identifier MODEL1310130000.

where *R* is the reaction rate, *V*_*max*_ is the maximum reaction rate, *K*_*m*_ is the Michaelis constant, and *c* is the concentration of the substrate.

The only exception from this kinetics law, is the passive transport of ethanolamine across the parasite membrane. This process has been modelled as a first order mass action law reaction as follows:

(2) $$R(c^{\text{ext}},c^{\text{in}})=K_{f}c^{\text{ext}}-K_{b}c^{\text{in}}$$

where *R* is the ethanolamine influx, *K*_*f*_, *K*_*b*_ are first order kinetic constants and *c*^*e* *x* *t*^, *c*^*i* *n*^, are the external and internal concentrations of ethanolamine, respectively.

The topology of the metabolic network is summarized by the stoichiometric matrix ***S***. The columns of ***S*** are the stoichiometric vectors, and the elements *S*_*ij*_ are integers representing the numbers of molecules of the species *i* that are consumed (in which case *S*_*ij*_<0) or produced (in which case *S*_*ij*_>0) by the reaction *j*. The ordinary differential equations describing the kinetics of the model read:

(3) $$\frac{dc^{\text{in}}}{\text{dt}}=SR(c^{\text{in}},c^{\text{ext}})$$

where ***R*** =(*R*_1_,*R*_2_,…,*R*_*r*_)^*T*^ is the column vector of reaction rates, in other words the vector of fluxes.

Given the external concentrations of precursors, ***c***^*e* *x* *t*^, the model can predict the intracellular distribution of fluxes, and the concentrations of metabolites, ***c***^*i* *n*^, as functions of time. These predictions are solutions of the Eq. (3) and depend on the kinetic parameters ***k*** =(*K*_*m*1_,*V*_*m* *a* *x*1_,…*K*_*m* *r*_,*V*_*m* *a* *x* *r*_,*K*_*f*_,*K*_*b*_) and on the external concentrations ***c***^*e* *x* *t*^. Rather generally, parameter fitting is a least-square optimisation problem. In our situation, the least-square objective function is

(4) $$\Phi (k)=\overset{d}{\underset{j=1}{\sum }}||F(k,c_{j}^{\text{ext}})-c_{j}^{\text{in}}|{|}^{2},$$

where the index *j*∈ [ 1,*d*] denotes several external concentration values $c_{j}^{\text{ext}}$ of the precursors, $c_{j}^{\text{in}}$ are the measured concentrations of metabolites after a long incorporation time (at steady state) and the vector function ***F*** gives the predicted metabolite concentrations. Here || ***x*** || stands for the Euclidean norm of the vector ***x***.

Of course, steady state data do not uniquely determine steady state parameters. Indeed, multiplying all the flux parameters (*V*_*max*_ in Michaelis-Menten kinetics) by the same constant, does not change the steady state and preserves the value of the objective function *Φ*. In order to fix this multiplicative constant, we used the values of the influxes, that were estimated by dividing the cumulated quantity of end products by the time needed for their accumulation.

Another, more difficult, problem is how to avoid local minima of *Φ*. In order to solve this problem we use a hybrid method combining discrete sampling of flux values, inversion of the smooth flux-concentration relation, and a final smooth local optimization. The main idea of this method can be summarized as follows. If for each reaction, we can determine the flux for various substrate concentrations, then we can invert the flux-concentration relation for the Michaelis-Menten mechanism and obtain *V*_*max*_ and *K*_*m*_. The optimisation of *V*_*max*_ and *K*_*m*_ has an unique solution, as can be shown by the well-known Lineweaver-Burk plot [43]. However, even though the substrate concentrations are readily available from the data, the flux profiles are unknown. A global optimum will be found by sampling the discretized space of admissible flux profiles.

From (3) we find that the steady state fluxes satisfy the equation

(5) SR=0.

Although it constrains the flux values, Eq. (5) has not an unique solution. The number of independent steady state flux profiles is equal to the dimension of the kernel *Ker*(***S***) of the stoichiometric matrix ***S***. The rank-nullity theorem provides the number of independent steady state flux profiles, *dim*(*Ker*(***S***))=*n*−*rank*(***S***), where *n* is the number of reactions in the network, and *rank*(***S***) is the rank of the matrix ***S***, i.e. the number of reactions that have linearly independent stoichiometries. According to the rank-nullity theorem, if there are *n* reactions in the network, there are *n*−*rank*(***S***) linearly independent, distinct flux profiles, compatible with the constraints. We call admissible fluxes, the solutions of Eq. (5) such that *R*_*i*_≥0, for any irreversible reaction.

In order to sample the set of admissible fluxes, we need a convenient parametrization of the admissible flux values. For simplicity, we present such a parametrisation for the case when the network contains only monomolecular reactions, i.e. each reaction has only one substrate and only one product. Our kinetic model satisfies this condition, because we chose not to represent cofactors. We also consider that all reactions are irreversible and impose *R*_*i*_≥0 for all fluxes. This condition is fulfilled by all reactions in our model, with the exception of the passive ethanolamine influx that is reversible. The later does not represent a problem, because in our data there is no ethanolamine incorporation and the corresponding reaction functions unidirectionally to evacuate internal ethanolamine excess. The generalization of the method to networks with reversible reactions is straightforward. In this case a bidirectional reaction can be replaced by two unidirectional reactions, each one having positive rate.

${\left\{R_{j}^{\text{in}}\right\}}_{j\;\in \;[1,n_{1}]}$ denotes the influxes,the fluxes that enter the network (in our case *n*_1_=3, there are three different PL precursors). Similarly, ${\left\{R_{j}^{\text{out}}\right\}}_{j\;\in \;[1,n_{2}]}$ denotes the effluxes, namely the fluxes that leave the network (in our case *n*_2_=3, the model produces three main PLs, namely PC, PE and PS). Given the influxes, the steady state flux distribution depends on a number of branching parameters $\alpha_{j}^{i}$, satisfying the relations $\underset{i}{\sum }\alpha_{i}^{j}=1,0\leq \alpha_{i}^{j}\leq 1$ and defined as follows. For each metabolite *j*, let us denote by ${\left\{R_{i}^{\text{out},j}\right\}}_{i\;\in \;[1,n_{j}^{\text{out}}]}$ the fluxes that consume the metabolite and by ${\left\{R_{i}^{\text{in},j}\right\}}_{i\;\in \;[1,n_{j}^{\text{in}}]}$ the fluxes that produce the metabolite. The corresponding stoichiometries (the numbers of molecules of metabolite *j* produced or consumed by the reaction *i*) are denoted by $\nu_{i}^{\text{in},j}>0$, $\nu_{i}^{\text{out},j}>0$, respectively. The positive integers $n_{j}^{\text{out}}$, $n_{j}^{\text{in}}$ will be called flux outdegree and indegree, respectively. For each metabolite *j* whose flux outdegree satisfies $n_{j}^{\text{out}}>1$, we define the positive parameters $\alpha_{i}^{j}$ such that $\overset{n_{j}^{\text{out}}}{\underset{i=1}{\sum }}\alpha_{i}^{j}=1$. Then, any admissible solution can be computed from the influxes by the following relations (see Additional file 1 for the proof):

(6) $$R_{i}^{\text{out},j}=[\;\overset{n_{j}^{\text{in}}}{\underset{k=1}{\sum }}R_{k}^{\text{in},j}\nu_{k}^{\text{in},j}/\nu_{i}^{\text{out},j}]\alpha_{i}^{j}$$

This reasoning also leads to the following formula for the nullity of a monomolecular network, which is the number of independent admissible flux profiles:

(7) $$\text{dim}(\text{Ker}(S))=n_{1}+\underset{j}{\sum }(n_{j}^{\text{out}}-1),$$

where *n*_1_ is the number of influxes. Eqs. (6), (7) show that in a network without branching (when all $n_{j}^{\text{out}}=1$), all the fluxes can be uniquely calculated from the influxes. Relations (6) are also applicable to non-monomolecular networks. However, the nullity formula (7) should be modified in general. Indeed, the same flux can consume several metabolites, which introduce further constraints in the system (6); the result will be a decrease of the nullity with respect to the monomolecular value (7).

For networks with branching, the parametrization (6) is used to sample admissible fluxes by choosing values of the branching parameters $\alpha_{i}^{j}=p_{i}^{j}/N$, where $p_{i}^{j},N$ are positive integers satisfying $\underset{i}{\sum }p_{i}^{j}=N,0\leq p_{i}^{j}<N$. For each choice of branching parameters $\alpha_{i}^{j}$, the reaction fluxes are computed for all the available concentrations, resulting from changing the external concentrations of the incorporated precursors. Then, the Michaelis-Menten flux-concentration relations are inverted for all the reactions independently, providing kinetic parameters. Because of the fitting errors, admissibility of the predicted fluxes is only approximate. Therefore, a second optimization step is needed, this time for all the reactions together. The kinetic parameters resulting from the inversion of Michaelis-Menten relations are used as initial guesses for a Levenberg-Marquardt local optimizer, minimizing the objective function *Φ* (4). This algorithm outputs optimal values of the kinetic parameters, for each initial choice of the branching parameters $\alpha_{i}^{j}$. Each of the resulting kinetic parameters ***k*** is a local minimum of the objective function *Φ*. By comparing these values of *Φ* one can find the global minimum.

**Figure 1:**
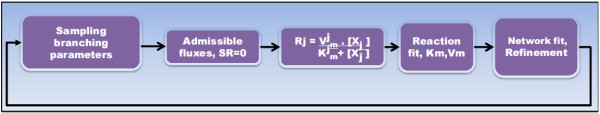
**Flowchart illustrating the hybrid optimisation method used for modeling.** Branching parameters are discretized and sampled to calculate admissible fluxes. For each individual reaction, Michaelis-Menten formula is inverted to obtain the parameters *K*_*m*_,*V*_*max*_. A final optimisation step leads to refined parameters for the full network.

In our method, the sampling of admissible fluxes can be exhaustive (for simple networks this is doable), or stochastic, using, for instance, simulated annealing in the discretized simplex of branching parameters. The flowchart of our optimisation procedure is represented in Figure 1.

### Multi-objective optimization

In order to analyse complex metabolic networks with a large number of parallel pathways one needs several types of fluxomic datasets, obtained in various conditions. In this paper we use two types of data corresponding each to incorporating only one PL precursor, either serine or choline. This leads to two datasets and least-squares objective functions. For simplicity of the calculations we combine the two objective functions by summing them. Because of the small overlap of the two datasets, more sophisticated analysis using Pareto optimality would lead to similar results.

### Flux balance analysis

Flux Balance analysis (FBA) is an alternate method to compute fluxes, given the reaction network and the biomass definition. It is based on the steady state constraint (5) and optimality of biomass production [44]. Throughout this paper we have used the following equation for the Biomass rate: 45% efflux PE + 50% efflux PC + 5% efflux PS is maximal [12,21]. FBA does not determine influxes, that can be arbitrarily normalized by multiplication with a constant. In order to compare FBA fluxes and values resulting from another method, the multiplicative constant should be chosen such that the influx, or, in the case of several influxes, the average input flux is the same in both methods.

### Limiting step determination

Although very popular among biochemists, the limiting step concept surprisingly lacks a clear definition [45]. Citing IUPAC Compendium of Chemical Terminology, rate-controlling, or rate-determining, or rate-limiting step in a reaction mechanism is an elementary reaction which exerts a strong effect - stronger than any rate constant - on the overall rate. The quantitative expression of this effect could be given by a sensitivity coefficient, defined as the derivative of the logarithm of the flux *F* with respect to the logarithm of the rate constant *k*:

(8) $$C_{F}^{k}=\frac{\partial \log(F)}{\partial \log k}.$$

In our study, the parameter *k* can be either *V*_*max*_ or *K*_*m*_. Also the flux value can be the steady state value, or, if steady state conditions can not be reached, the value at a given time, suggested by experiment. Let us notice that our sensitivity coefficients become the flux control coefficients from metabolic control theory, only when *k*=*V*_*max*_ and *F* is the steady state flux. In this case only, *F* is homogeneous in the parameters and the corresponding control coefficients satisfy the usual summation theorems [46]. As discussed by Ray [47], the use of sensitivity analysis in this context can lead to existence of many important reactions instead of just one limiting step. In other words, one can speak of limiting steps when sensitivities are concentrated (there is one or a few important reactions) instead of dispersed (all the reactions are equally important) [48]. The concept of limiting step is often assimilated to a slow step or narrow place in a chain of transformations. This choice has a meaning for linear pathways, but has to be revised for pathways with branching and cycles [45]. Furthermore, in a simple chain of transformations, the steady state flux, common to all the reactions in the chain, is controlled by the rate constant of the first reaction and does not depend on parameters of other reactions. Metabolic control leads to a trivial result in this case : irrespectively of the presence or not of a narrow place, the flux control coefficients are all zero, excepting for the first step that has control coefficient one. However, a narrow place in a chain of transformations is limiting in the sense that it provides an upper bound to the steady state flux. One gets unlimited accumulation of downstream metabolites, if the narrow place is not the first step of the chain and if the conduction capacity of the narrow place is exceeded.

## Results and discussion

### Modeling the structural phospholipid (PL) synthetic pathways in *Plasmodium knowlesi*

**Figure 2:**
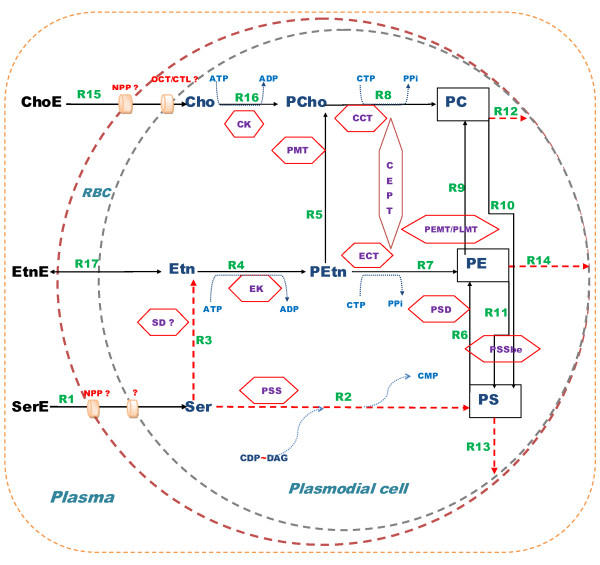
**Schematic overview of*P. knowlesi* reactions in structural phospholipid biosynthesis as demonstrated by experimental work.** Solid lines correspond to metabolite transport or biochemical reactions with an identified enzyme whereas dotted line correspond to reaction identified with unidentified enzyme. Squares represent the phospholipid end products. Hexagons represent the enzymes. R1 to R17 denote the reaction rates/fluxes. List of species : SerE = exogenous serine, Ser = intracellular serine, PS = phosphatidylserine, EtnE = exogenous ethanolamine, Etn = intracellullar ethanolamine, PEtn = phosphoethanolamine, PE = phosphatidylethanolamine, ChoE = exogenous choline, Cho = intracellular choline, PCho = phosphocholine, PC = phosphatidylcholine,DAG = diacylglycerol, SD = serine decarboxylase, PSSbe = phosphatidylserine synthase I, PMT = phosphoethanolamine-N-methyltransferase, PEMT = phosphophatidylethanolamine-N-methyltransferase, CCT = cholinephosphate cytidylyl transferase, ECT = ethanolaminephosphate cytidylyl transferase, CEPT = choline/ethanolamine phosphotransferase, CK = choline kinase, EK = ethanolamine kinase, NPP = New permeation pathway, OCT = Organic cationic transporter, ? = putative genes found.

Phospholipid synthesis in *P. knowlesi* parasite at its blood stage is one of the most characterized metabolic network, due to the availability of infected erythrocyte collected from *Macaca mulatta* or *M. fascicularis* monkeys and several thorough fluxomic studies [28,49,50]. Figure 2 represents the network of reactions that are supported by biochemical findings. It provides a global overview of the pathways present in *P. knowlesi*. The availability of the genome sequence of *P. knowlesi* and the subsequent genomic annotations brought a considerable amount of information for the existence of biological processes and existing biochemical pathways, offering a global view of the parasite biology.

Lipid metabolism in *Plasmodium knowlesi* takes place to a higher extent in late trophozoite and early schizont stage of asexual (erythrocytic) phase [28,49].

The intra-erythrocytic proliferation of *P. knowlesi* requires large amount of PC and PE that together constitute the bulk of the malaria membrane lipids [12]. The very high biosynthetic capacity of *Plasmodium* operates at the expense of the fatty acids mainly originating from the plasma and polar heads building units. Choline entry into infected red blood cells (IRBC) involves the erythrocytic choline carrier [38,51] and parasite-induced new permeation pathways (NPP) [20,38-40]. Choline is provided to the parasite by a characterized and very efficient organic-cation transporter (OCT/CTL) [20,38-40].

Ethanolamine can be supplied from the poorly available plasmatic ethanolamine, which can cross the membrane by passive diffusion, and from serine. Serine is diverted from the serum, host RBC or from hemoglobin degradation in food vacuoles [40].

#### Kennedy pathways

Both PC and PE may be synthesized denovo by the CDP-choline or CDP-ethanolamine-dependent Kennedy pathways. In the so-called Kennedy pathway, choline is phosphorylated into phosphocholine (PCho), which is subsequently coupled to CTP, thus generating CDP-choline, which is further converted to PC by a parasite CDP-diacylglycerol-cholinephosphotransferase (CEPT). A similar de novo pathway allows the synthesis of PE from ethanolamine. The final stage of both branches of the Kennedy pathway involves the same CEPT enzyme, catalyzing the formation of PC and PE from CDP-choline and CDP-ethanolamine, respectively [21,23,33].

#### CDP-DAG pathways

Additionally, *Plasmodium* possesses the CDP-DAG-dependent ancestral pathway, which provides the anionic phospholipids PI, PG, cardiolipid and eventually PS [9]. Biosynthesized PS is converted into PE via the activity of PS decarboxylase (PSD) [32].

#### SDPM plant-like horizontal pathways

*P. knowlesi* also possess a plant-like pathway that relies on serine to provide additional PC and PE, which is named the serine decarboxylase-phosphoethanolamine methyltransferase (SDPM) pathway. Hereby, serine is first decarboxylated to form ethanolamine, which is then phosphorylated to lead to phosphoethanolamine (PEtn). Serine decarboxylase (SD) enzymatic activity was first described by our group in *P. knowlesi* and *P. falciparum*[28]. The gene and related SD catalytic activities were subsequently identified in plants [52]. The resulting phosphoethanolamine is either incorporated into PE via the CDP-ethanolamine pathway or converted into phosphocholine by SAM-dependent triple methylation, which is carried out by a plant-like phosphoethanolamine N-methyltransferase (PfPMT) (EC 2.1.1.103) [30,31]. In the Apicomplexa phylum, the SDPM-pathway is only conserved in Plasmodia with the exception of rodent parasites, where the PMT activity is absent [53].

#### Base-exchange reactions

Base-exchange reactions (PSSbe) between serine and PE or PC were initially not detected in *Plasmodium*. However, parasite genomes suggest corresponding hypothetic gene and recent biochemical studies have revealed calcium-dependent base exchanges between serine and PLs in *P. falciparum* (Berry and Vial, unpublished data). There is no data measuring their quantitative importance but they would be operational at the erythrocytic stage. In some plants species, in which both CDP-DAG-dependent and base-exchange PS synthesis take place, it has been shown that, these enzymatic reactions have different preferential molecular species as substrates [54].

Glycerophospholipid model (PL model) shown in Figure 2 represents a rather exhaustive scheme encompassing the phospholipid synthesis and metabolic reactions in *Plasmodium*, including 17 reactions and enzymes. Biochemical experimental data and quantification experiments, supports that this parasite machinery can provide the bulk of PL composing the *P. knowlesi* membranes. Genomic studies have confirmed that malaria parasites possess most of the panoply of corresponding genes (see Table 1). Some of the genes, such as those coding for the base exchange and for PS synthase (PSS) enzymes, remain hypothetical in *Plasmodium*. One of the aims of our quantitative modelling is to test the relative importance of various reactions in the model and, eventually, the absence for activity of some of them.

#### Model simplification

In CDP-choline pathway, PC is produced from PCho in two steps. First, PCho gives CDP-choline and then CDP-choline transforms to PC. The intermediate CDP-choline was produced in minute quantity which was difficult to measure experimentally [28,53]. Again the yield of labelled PCho was found to be 35 times more as compared to CDP-choline. Thus, the formation of PC from CDP-Choline is very rapid relative to the formation of CDP-choline from PCho [28] and CDP-choline is a quasi-steady state species. So, we have ignored this fast intermediate and combined the 2 reactions into a single reaction (labelled R8 in the model).

Similarly, in CDP-ethanolamine pathway formation of PE from PEtn takes place in two steps. PEtn forms CDP-ethanolamine and in turn produces PE. Again CDP-ethanolamine is produced in minute quantity which is not possible to quantify experimentally. Formation of PE from CDP-ethanolamine is very rapid with respect to the rate limiting formation of CDP-ethanolamine from PCho [50]. So, we have combined these two reactions into a single reaction (labelled R7).

### Training the model

The glycerophospholipid model (PL model) was trained with two datasets, (i) incorporation of serine [28] (ii) incorporation of choline [50] to their different metabolites (PS,PE,PC) in the phospholipid metabolism pathway. The experimental datasets includes the steady state concentrations of the radiolabelled precursors (serine and choline) with respect to their exogenous concentrations.

#### PL model trained with serine and choline incorporation datasets

**Figure 3:**
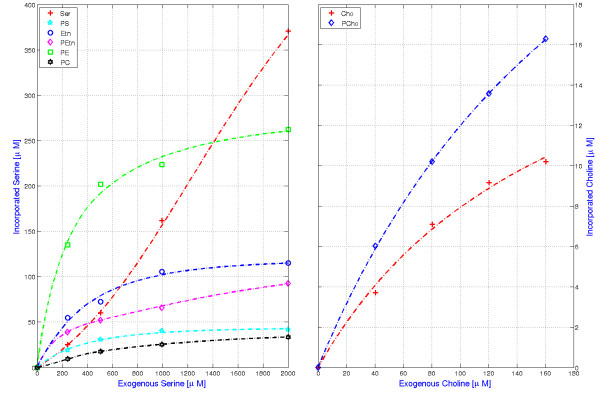
**The plot illustrates the fit between the steady state concentration of serine and choline incorporated metabolites with the extracellular serine (0–2000*μM*)(left panel) and choline (0–160*μM*)(right panel) from experimental studies and from simulation respectively.** The points(or markers) represent the steady state concentration of serine incorporated metabolites with varying extracellular serine measured in the experiment. The dash-lines represent the concentration of metabolites with varying concentration of extracellular serine, as calculated from (PL) Model(ODE).Similarly, in the right panel, points(or markers) represent the steady state concentration of choline incorporated metabolites with varying extracellular choline measured in the experiment. The dash-lines represent the concentration of metabolites with varying concentration of extracellular choline, as calculated from (PL) Model(ODE).

In the experiment [28], variable amounts from 0 to 2000 *μM* of radiolabelled serine were subjected exogenously to the cell and were incorporated into various metabolites (see Figures 2 and 3). These data was used to train the model. The steady state concentration of all the serine derived metabolites were predicted and used to fit the experimental data by the procedure defined in the Methods section. For each extracellular serine concentration, the influx of serine was calculated by dividing the total amount of accumulated end products (PC + PE + PS) by the characteristic accumulation time (2 h).

The characteristic accumulation time correspond to the cross-over between accumulation that is approximately linear in time and corresponds to constant net fluxes, and saturation, corresponding to proximity of steady state (when the net fluxes vanish). For serine incorporation, this time is approximately two hours [28].

Radiolabelled experiments were also performed with choline as metabolic precursors. Choline produces different metabolic compounds (see Figures 2 and 3) with PC as the major end product [50]. This dataset was also used to train the model, with a unique set of parameters for both experiments. In order to reproduce the experimental protocol, extracellular concentration of choline (ChoE) was taken for several values from 0 to 160 *μM*, whereas concentrations of extracellular serine (SerE) and ethanolamine (EtnE) were kept very low. Like for the previous data sets, the influx of precursor was calculated by dividing the total amount of accumulated end product (PC) by the accumulation time (1 h). Though used for influx estimates, the phosphatidylcholine does not reach steady state in this experiment (see Figure one of [50], also the model analysis section) and PC profile has not be taken into account for the calculation of the objective function *Φ*, defined by Eq. (4).

A model prediction with the experimental results is shown in Figure 3. The corresponding parameter values can be found in Table 2.

**Table 2:** Parameter values and confidence intervals for the ODE (PL) model

**Reaction ID**	**Reactions**	**Enzymes**	**Estimated Vm( *μ M */min)**	**Estimated Km ( *μ M *)**	**Regime**
R1	SerE → Ser	..	3.41∈ [ 3.08,3.95]	363∈ [ 326,410]	
R2	Ser → PS	PSS	1.31∈ [ 0.517,1.65]	797∈ [ 352,1.25.10^3^]	
R3	Ser → Etn	SD	2.62∈ [ 2.33,3.11]	24∈ [ 22.7,29.5]	
R4	Etn → PEtn	EK	8.62∈ [ 6.19,13.3]	109∈ [ 75.6,193]	
R5	PEtn → PCho	PMT	1.08∈ [ 0.725,13.9]	122∈ [ 78.5,2650]	
R6	PS → PE	PSD	2.25∈ [ 0.747,2.94]	204∈ [ 152,413]	
R7	PEtn → PE	ECT/CEPT	5.61∈ [ 4.19,7.65]	227∈ [ 175,353]	
R8	PCho → PC	CCT/CEPT	0.413∈ [ 0.315,0.599]	31∈ [ 26.2,34.9]	
R9	PE → PC	PEMT/PLMT	1.42.10^3^∈ [ 59.2,1.33.10^4^]	3.21.10^5^∈ [ 2.73.10^4^,1.92.10^6^]	L
R10	PC → PS	PSSbe	0.697∈ [ 0.318,0.944]	3.76∈ [ 0.06,3.88]	
R11	PE → PS	PSSbe	89.9∈ [ 9.16,527]	1.71.10^5^∈ [ 1.58.10^4^,9.14.10^5^]	L
R12	PC →	..	1.57∈ [ 0.75,3.84]	24.1∈ [ 16.5,35.8]	
R13	PS →	..	1.54∈ [ 0.013,3.28]	204∈ [ 41.1,1020]	
R14	PE →	..	774∈ [ 9.44,1.08.10^4^]	1.55.10^5^∈ [ 2.52.10^4^,1.65.10^6^]	L
R15	ChoE → Cho	OCT/NPP	0.232∈ [ 0.182,0.323]	102∈ [ 91.9,109]	
R16	Cho → PCho	CK	0.556∈ [ 0.384,0.882]	30.4∈ [ 22.8,36.7]	
R17	EtnE → Etn	..	*K*_*f*_=5.10^−4^*min*^−1^∈ [ 10^−4^,2.5.10^−3^]	*K*_*b*_=1.33.10^−4^*min*^−1^∈ [ 4.45.10^−15^,1.4.10^−3^]	

For the calculation of the confidence intervals see the Methods section and the Additional file 1 (the allowed objective function values were less than 1.5 times the global minimum). All the reactions have mass action kinetics, with the exception of the reaction 17 that has first order mass action law kinetics (the forward and the backward rate constants are denoted *K*_*f*_ and *K*_*b*_, respectively). For those reactions whose regime is linear (L), only the ratio *V*_*max*_/*K*_*m*_ is significant.

### Model predictions and analysis

#### Calculations of fluxes from the model (ODE method) and comparison with Flux Balance Analysis (FBA)

Using the parametrized ODE model we can compute the steady state concentrations of the metabolites, as well as the steady state values of the fluxes through all reactions. Steady state fluxes can also be calculated using the FBA method (see Methods). We compare the results from two methods. Because FBA does not fix the time scale, in this method fluxes are determined up to multiplication by a constant. In order to compare the two methods, we renormalized the FBA fluxes such that the input fluxes (the average when there are several) coincide in the two methods. Although there is no reason to expect that the FBA method provides an absolute reference, this comparison is informative. Agreement will confirm the relevance of the optimal biomass production concept, whereas disagreement will indicate the limitations of the FBA method.

We have performed different *in silico* experiments with the incorporation of different metabolic precursors.

##### Serine incorporation

**Figure 4:**
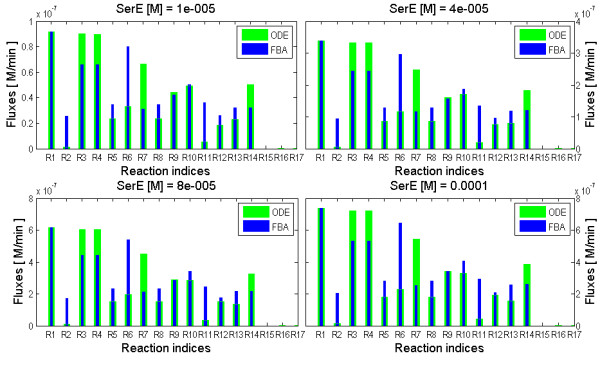
**The bar plots shows the distribution of fluxes in the network with four different concentrations of SerE [0-100*μ*M].** In each subplot the reaction rates/fluxes are denoted by (R1 to R17). The blue bar color represent fluxes from the FBA method and green bars represent fluxes from ODE.

At physiological concentration of exogenous serine (SerE) (0-100 *μM*), fluxes were calculated using the FBA method [44] and the ODE kinetic model (with fitted parameters). The fluxes from these two different methods were compared. The concentration of extracellular choline (ChoE) and ethanolamine (EtnE) was kept very low. Distribution of fluxes with five different concentration of SerE (0-100 *μM*) is shown in Figure 4.

Fluxes from FBA (represented with blue bars) are independent of the concentration of the metabolites but they depend on the assigned biomass and on the influxes. The biomass was defined to be (50% PC + 5% PS + 45% PE) [12,21].

Relative to the influx, the distribution of fluxes from FBA method (blue bars, Figure 4) does not change with the concentration of exogenous serine (SerE). On the other hand, the relative distribution of fluxes calculated by the ODE method, changes slightly for some reactions with change in concentration of SerE. The fluxes from both the methods were compared to each other and to previously reported biochemical findings.

Fluxes from ODE (green bars, Figure 4) marks the major part of serine derived PE, formed via serine decarboxylation (SD)(R3 in Figure 2 and Figure 4). PS decarboxylation (PSD)(R6 in Figure 2 and Figure 4) which also can form PE, has less contribution.

Indeed, the flux R7 is much higher than R6 and it does not increase much with increase in concentration of exogenous serine (SerE, Figure 4). Again, R3 is much greater than R2. A very low flux R2 indicates low, possibly vanishing, activity of phosphatidylserine synthase (PSS) during blood stages of *P. knowlesi* in presence of exogenous serine.

These findings suggest that, direct decarboxylation of serine and CDP-ethanolamine pathway (R3,R4,R7) (see Figure 2) is the preferred pathway for the formation of PE from serine in agreement with [28].

On the contrary, fluxes from FBA method (blue bars, Figure 4) suggest that R6 is greater than R7. So, the FBA results contradict the experimental findings [28]. This contradiction may be due to the lack of relevance of the biomass optimisation in the situation when serine only is incorporated and emphasizes the utility of kinetic modeling.

Base-exchange reactions (PSSbe) between serine and PE or PC were initially not detected in *Plasmodium*. However, recent biochemical studies revealed calcium-dependent base exchanges between serine and PLs in *P. falciparum (Berry and Vial, unpublished data)*. This is an area which remains unexplored in *Plasmodium*.

PS could be formed directly from serine via PS synthase (R2), or by transformation of PC and PE via (PSSbe), R10 and R11 respectively (Figure 2). In order to understand the relative importance of these pathways, we compared the fluxes R2, R10 and R11 in both the methods.

Fluxes from ODE suggest that, very less quantity of PS was produced from serine via R2 (PSS) but considerable amount from PC (R10) and PE (R11). The flux values of R2 from FBA (blue bars) was much higher than the ODE (green bars), though remained significantly smaller than R3. It means considerable amount of PS was formed from serine via R2 (PSS), which quantitatively contradicts the biochemical findings [28]. However, the flux R10 (PSSbe) from both the methods suggests an exchange of PC and PS in presence of SerE.

### Source of PC production in absence of CDP-choline pathway

The major source of PC is thought to be provided by CDP-choline or Kennedy’s pathway [50,55]. Thus, the denovo CDP-choline pathway has been proposed to be the primary route for synthesis of PC in *Plasmodium*[50,56]. However, *in vitro* growth assays using dialyzed serum indicated that CDP-choline pathway was not essential for parasite intra-erythrocytic development and survival [57,58]. There are two possible pathways which could furnish PC synthesis other than CDP-choline pathway/Kennedy pathway (see Figure 2): 

i. PMT, which transforms PEtn into PCho, which in turn forms PC via CDP-choline pathway. This is a part of SDPM pathway by which host serine is incorporated into PC. The mechanism is S-adenosyl methionine (SAM)-dependent triple methylation carried out by a plant-like phosphoethanolamine N-methyltransferase (PMT or PEAMT) (EC 2.1.1.103).*P. falciparum* PMT (PfPMT), has been revealed by the *P. falciparum* genome sequencing program [59]. Subsequently, the role of phosphoethanolamine methyltransferase (PMT) pathway has been identified in *Plasmodium falciparum*[31]. The orthologous gene (PKH_121150) has been identified in *Plasmodium knowlesi*[36].

ii. PE transmethylase (PEMT or PLMT) pathway (R9 in Figure 2). The capacity of *P. knowlesi*-infected erythrocyte to methylate PE into PC has been documented in previous studies and clearly indicates PEMT activity [37]. However the corresponding genes have not yet been found in any *Plasmodium* species.

It is thus necessary to understand the kinetics of SDPM and PE transmethylase pathway which diverts host serine and ethanolamine (via PE) into PC. For this, we compared the fluxes R5 (PMT) and R9 (PEMT/PLMT) from ODE and FBA methods (Figure 4). We found that behavior of the fluxes in both the methods is coherent. Flux R9 (PEMT/PLMT) is slightly more as compared to R5 (PMT) in both FBA and ODE methods. These suggests that both R5 and R9 could act as an source for the production of PC.

In order to gain further understanding into the relevance of R5 (PMT) or R9 (PEMT/PLMT) *in silico* knockout experiments were performed.

#### In silico knockout of R5 (PMT), phosphoethanolamine-N-methyltransferase

**Figure 5:**
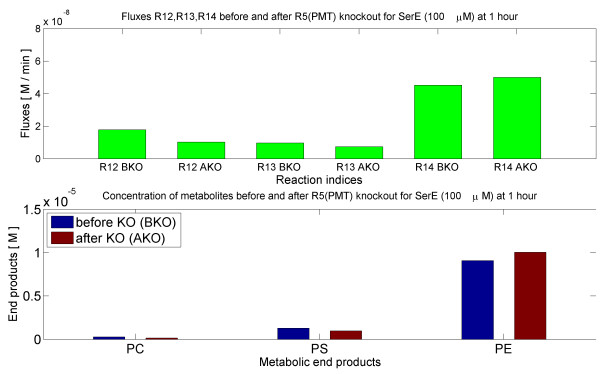
***In silico* knock-out of PMT (R5).** The bar plots illustrate the results of *in silico* knock-out of PMT in presence of SerE (100 *μM*) and in absence of ChoE, EtnE. ‘BKO’ denotes before knockout and ‘AKO’ denotes after knockout. The upper panel shows the effect on effluxes, whereas the lower panel shows the effect on concentrations of end products. The fluxes and concentrations were calculated by simulating the ODE (PL) model for two hours starting from zero initial metabolite concentrations.

The PL Model was simulated for two hours with the knockout of R5(PMT). The exogenous serine (SerE) was kept at 100 *μM*, whereas the concentrations of choline and ethanolamine were kept very low. The PC efflux (R12), PS efflux (R13) and PE efflux (R14) were estimated before knockout (BKO) and after knockout (AKO) as shown in Figure 5.

The result is a two fold decrease in the efflux R12 and PC concentration (see Figure 5). Thus, when R5(PMT) was knocked out, the rate of incorporation of PC into the membrane decreased in the absence of exogenous choline (ChoE). However, there was a small increase in the rate of PE efflux.

#### In silico knockout of R9 (PEMT/PLMT), phosphatidylethanolamine-N-methyltransferase

**Figure 6:**
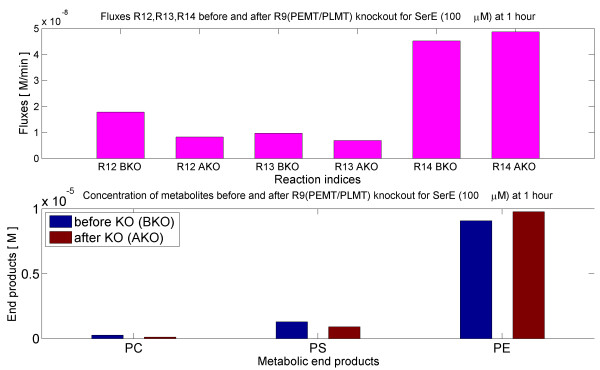
***In silico* knock-out of PEMT/PLMT (R9).** The bar plots illustrate the results of *in silico* knock-out of PEMT/PLMT in presence of SerE (100 *μM*) and in absence of ChoE, EtnE. ‘BKO’ denotes before knockout and ‘AKO’ denotes after knockout. The upper panel shows the effect on effluxes, whereas the lower panel shows the effect on concentrations of end products. The fluxes and concentrations were calculated by simulating the ODE (PL) model for two hours starting from zero initial metabolite concentrations.

Similarly, the PL Model was simulated for two hours with the knockout of R9 (PEMT/PLMT). The fluxes before knockout (BKO) and after knockout (AKO) are shown in Figure 6.

There was a marked decrease in PC efflux (R12) and concentration. Thus, when R9 (PEMT/PLMT) was knocked out, the rate of incorporation of PC into the membrane decreased in the absence of exogenous choline (ChoE).

The behavior of fluxes with R5 (PMT) or R9 (PEMT/PLMT) knockout followed the same pattern. There was a significant decrease in PC efflux (R12), and a small increase in the PE efflux (R14). A marked R9 (PEMT/PLMT) reaction denotes the capacity of the parasite to convert PE to PC.

From the knockout studies (see Figure 5 and Figure 6) it was found that, part of PC was produced by the even contribution of R5(PMT) and R9(PEMT/PLMT). Looking to the results of [30,31,60], knockout of PMT pathway in P.falciparum produces a severely affected phenotype. This suggests that PC biosynthesis from SPDM pathway cannot be compensated by the CDP-choline pathway. This is only partially in agreement with our predictions and could mean that PMT might also have PEMT activity *in vivo* in particular conditions. Because the corresponding genes coding for PEMT have not been found in any *Plasmodium* species, deletion of PMT gene in *P. falciparum* could be equivalent to a double knockout of PMT and PEMT in our model, that completely abolishes the incorporation of ethanolamine into PC.

### Rate limiting steps for PC synthesis

We are interested here in detecting rate limiting steps for the major PC synthesis pathway, namely the CDP-choline pathway. The analysis is based on experiments that have been done with choline as unique metabolic precursor [50]. This experimental setting was designed to probe the de novo synthesis of PC via CDP-choline pathway. In this experiments the extracellular concentration of choline (ChoE) was changed from 0 to 100 *μMin silico*. Concentrations of extracellular serine (SerE) and ethanolamine (EtnE) were kept very low. As shown in Figure one of [50], the parasite choline and phosphocholine reach steady state concentrations after two hours of incorporation. However, the phosphatidylcholine concentration is linearly growing with time even after three hours of incorporation and does not reach steady state. The incorporation data correspond to concentration of choline derived metabolites after one hour of incubation of infected erythrocytes in the presence of radiolabelled choline. It explains the dynamics of the pathways to provide PC which is readily incorporated into *P. knowlesi* membrane or structural phospholipids.

We have simulated the ODE model for 1 h starting with vanishing initial metabolite concentrations and computed the resulting fluxes. The kinetic parameters (*K*_*m*_, *V*_*max*_) are the ones obtained from model training and common both to serine and to choline incorporation.

In order to find limiting steps we use a sensitivity based approach. Parameters *K*_*m*_, *V*_*max*_ are perturbed with respect to the nominal values. We compute sensitivity coefficients defined as the derivatives of the fluxes with respect to all parameters of the model (see Methods section).

**Figure 7:**
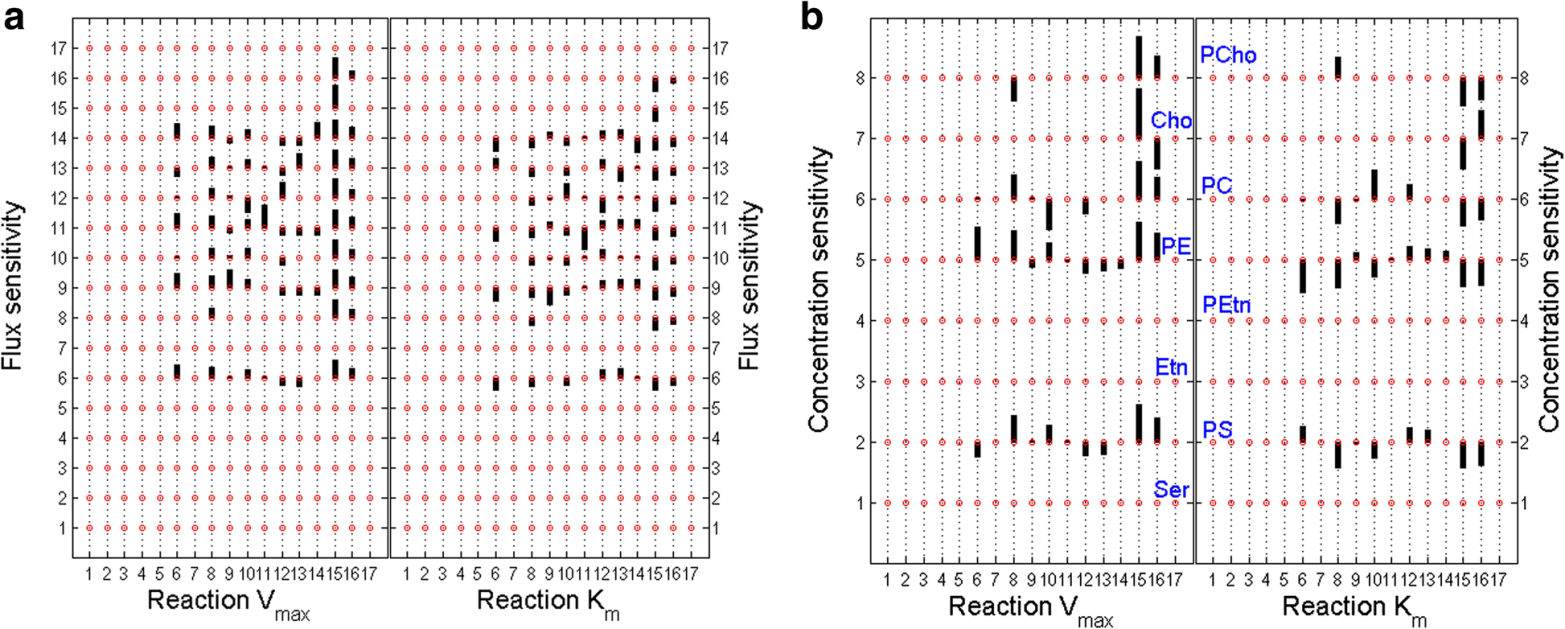
**The plots represent the result of the sensitivity analysis.** We have computed the sensitivity coefficients matrix for the fluxes (**panel a**) and for the metabolite concentrations (**panel b**) with respect to parameters *V*_*max*_ and *K*_*m*_. We have considered choline incorporation via Kennedy pathway, namely *ChoE*=50 *μM*, *SerE*=0, *EtnE*=0. Fluxes and parameters are numbered from 1 to 17 that correspond to reaction labels from Figure 2. Metabolites are numbered from 1 to 8, which corresponds to species represented in Figure 2. Each bar has a size proportional to the value of the control coefficient (when this coefficient is negative the bars are oriented downwards). We can notice that the strongest effect on both the PC production (flux 8, **panel a**) and PC concentrations (species 6, **panel b**) is produced by changes of parameters of reactions 15 and 8, in this order. However, all the reactions in the CDP-choline pathway (with the exception of the fast, non-represented one, transforming CDP-choline into PC) have comparable sensitivity coefficients, meaning that this pathway has not an unique limiting step. The PC concentrations are also sensitive to the base exchange reaction R10 (**panel b**), which is normal, because this reaction consumes PC.

As seen in Figure 7, for choline incorporation via the CDP-choline (Kennedy) pathway, sensitivity coefficients have similar orders of magnitude for fluxes and concentrations. These coefficients have even, rather than concentrated distributions.

Consequently, and contrary to our former study [50], the model cannot identify *stricto sensu* rate limiting steps. As expected, rate constants of reaction 15 (the choline influx) controls all the non-vanishing fluxes and concentrations. In order of importance,follows reaction R8 (CCT: phosphocholine cytidylyltransferase) and to a lesser extent, R16 (CK: choline kinase).

Our former experimental work [50]which finds CCT as rate-limiting step should be interpreted in different terms. Indeed, the relatively slow step CCT produces the quasi-steady state CDP-choline that is rapidly consumed and hence present in minute quantities compared to the CCT substrate PCho. This means that CDP-choline is a fast intermediate that can be depleted from the model, which we actually did from the very beginning when we reduced the model. The sensitivity coefficients of the remaining reactions, although different, do not differ by orders of magnitude.

## Conclusion

Precise quantitative models for essential metabolic functions of pathogens can be used to understand their intricate set of biological process and their regulation. It guides us to determine the specificities of their physiology, improve the action of known drugs and discover new treatments directed against them. Even when the full genome is available, as in the case of *Plasmodium*, the reconstruction of metabolic pathways can be particularly challenging. Generic pathways databases provide incomplete or unverified metabolic pathways for *Plasmodium*. For instance, Kegg database [61] proposes the similar glycerophospholipid metabolism map for *P. knowlesi*, *P. berghei*, and *Bacillus subtilis*, in fact based on evidence coming mainly from *S. cerevisiae* and *E. coli*. Hagai Ginsburg’s Malaria Parasite Metabolic Pathways (http://priweb.cc.huji.ac.il/malaria/) is more specific because based on data from *Plasmodium* species (without interspecies distinction), but is still incomplete and not quantitative. We have proposed here, for the first time, a complete quantitative model for the glycerophospholipid synthesis in *Plasmodium knowlesi*. This model is based on several fluxomic experiments of incorporation of radiolabelled phospholipid precursors.

In order to learn the metabolic network from data we have developed a new hybrid optimisation scheme, which is based on the discretization of the simplex that parametrizes the set of directions in the cone of admissible fluxes. The main interest of this method is that it facilitates the global search in the parameter space and can be combined with other global optimization algorithms, such as genetic algorithms or simulated annealing. Our method was specifically designed to understand glycerophospholipid metabolism in *Plasmodium* from radiolabelled precursor fluxomic data. It provides an effective sampling of the parameter space. This method can be generally applied to metabolic networks with Michaelis Menten reactions, functioning at steady state. It can be therefore used for other studies of the same kind, for rate constant identification in isotope labelling experiments.

The metabolic network model has been used to elucidate the functioning of the multiple phospholipid synthetic pathways in *P. knowlesi*. The main source of PC is the CDP-choline Kennedy pathway, however, SDPM and PE transmethylase pathways could provide part of PC. The values of the fluxes as well as *in silico* knock-out experiments showed comparable importance of PMT and PEMT/PLMT for PC synthesis in *P. knowlesi*.

These findings confirmed earlier hypotheses about the existence of both PMT and PEMT activity in *P. falciparum* and *P. knowlesi*[37]. Our *in silico* knock-out experiments prove partial dependence of PC production on both PMT and PEMT, meaning that single knock-out of any of these enzymes will reduce but not completely eliminate PC production from serine in *P. knowlesi*. This prediction can not explain the result of [60] that deletion of pfPMT gene in *P. falciparum* abolish the incorporation of ethanolamine into PC. Because the corresponding genes coding for PEMT have not been found in any *Plasmodium* species, altogether these findings could suggest that PfPMT might also have PEMT activity *in vivo* in particular conditions (which are not met *in vitro* or in yeast) [53,62].

Our model also indicate that the major part of serine derived PE is formed by serine decarboxylation. PS is predominantly formed by base-exchange reactions and not by the direct CDP-DAG phosphatidyl-synthase (PSS) mechanism.

Sensitivity analysis of CDP-choline pathway in our model, does not identify limiting steps. However, it shows that the carrier-mediated choline entry into the parasite and the phosphocholine cytidylytransferase (CCT) reaction have, in order, the largest sensitivity coefficients in this pathway. This finding is in agreement with previous knowledge, and has been partially exploited in the search for antimalarial drugs. Indeed, choline entry is targeted by a new the class of potent antimalarial drugs [19,63,64]. It would be interesting to combine this action with simultaneous inhibition of the CCT reaction.

## Competing interests

The authors declare that they have no competing interests.

## Authors’ contributions

PS, HV, and OR wrote the paper. PS and OR developed and implemented the methodology. All authors read and approved the final manuscript.

## Supplementary Material

Additional file 1 Proofs of mathematical results and calculation of the parameter confidence intervals. Click here for file
