# Supplementary material for: Kinetic modelling of phospholipid synthesis in Plasmodium knowlesi unravels crucial steps and relative importance of multiple pathways
Source: BMC Syst Biol. 2013 Nov 9;7:123. doi: 10.1186/1752-0509-7-123 (PMC3829661; doi:10.1186/1752-0509-7-123)
Supplement: Additional file 1 — Proofs of mathematical results and calculation of the parameter confidence intervals. [file 1752-0509-7-123-S1.pdf]

Additional file 1

Proofs of mathematical results, parameter confidence intervals, sensitivity analysis

Derivation of Eq.(6)

Let us consider a monomolecular reaction network. If some reactions are reversible, we split these reactions into two by using distinct indices for the forward and for the backward directions, in such a way that $R_i \geq 0$, for all i. Then, we show that the steady state condition defining admissible fluxes

$$\boldsymbol{S}\boldsymbol{R} = 0, \qquad (S1)$$

is equivalent to the branching parametrisation relation

$$R_i^{out,j} = [\sum_{k=1}^{n_j^{in}} R_k^{in,j} \nu_k^{in,j} / \nu_i^{out,j}] \alpha_i^j, \qquad (S2)$$

where $\sum_i \alpha_i^j = 1, 0 \leq \alpha_i^j \leq 1$.

Indeed, let us notice that $\nu_i^{out,j} = -S_{ji}$ and $R_i = R_i^{out,j}$, if $S_{ji} < 0$. Similarly, $\nu_i^{in,j} = S_{ji}$ and $R_i = R_i^{in,j}$, if $S_{ji} > 0$. Relation (S1) is equivalent to

$$\sum_{i, S_{ji}>0} S_{ji} R_i + \sum_{i, S_{ji}<0} S_{ji} R_i = 0,$$

that reads

$$\sum_{k=1}^{n_j^{in}} \nu_k^{in,j} R_k^{in,j} - \sum_{i=1}^{n_j^{out}} \nu_i^{out,j} R_i^{out,j} = 0 \qquad (S3)$$

The fluxes $R_i^{out,j}$ can always be expressed as in (S2), for some positive parameters α_i^j. However, if (S3) is satisfied, then $\sum_i \alpha_i^j = 1$ follows. Conversely, if (S2) is satisfied with $\sum_i \alpha_i^j = 1$, then (S3) follows.

Parameter confidence intervals

The available data constrain the values of many parameters. However, not all parameters are identifiable with the same precision. We describe here a simple post-processing procedure that allows to quantify the parametric uncertainty.

Our optimization method generates a set of values of parameters, that are local minima of the objective function. The lowest of such minima is the global optimum. To take into account possible experimental errors, and because several local minima can fit the experimental data reasonably well, we have considered not only the globally best fit, but also several local optima closest to the global optimum. Among these sets of parameters we considered as representative the one that is closest in the Euclidean distance to the median parameter value. The spread of parameter values around the median value provides a first estimate of the parameter range. Then, we have performed a local sensitivity analysis around each of the selected local minima of the objective function. To this aim, we multiply each parameter by factors that are independent, and log-uniformly distributed, namely $k_i = k_{0i} \exp((2U_i - 1)a)$, where U_i are independent, uniformly distributed in the interval $[0, 1]$, $a > 0$, and k_{0i}, k_i are the local optimum and the perturbed parameter values, respectively. The parameter range is defined in this case by all the perturbed parameters values k such that $|\log(\Phi(\boldsymbol{k})) - \log(\Phi(\boldsymbol{k}_0))| < \epsilon$, where ϵ is a small positive value. We call this perturbation scheme *uncorrelated, multiplicative.*

For further insight into the parametric uncertainty we have investigated, for each Michaelis-Menten reaction, the dynamical range of substrate concentrations. These ranges should ideally include concentration values both below and above the Michaelis constant K_m. In this case, both V_{max} and K_m are accurately determined. If the substrate concentration range lies below K_m, then the enzymatic reaction performs in its linear regime. In this case, one gets the ratio V_{max}/K_m, but V_{max} and K_m can not

be independently determined. If the concentration range is above K_m, then V_{max} is well determined, but for K_m one has only upper bounds. The situations corresponding to various parameters in this model are presented in Figure.S1.

For Michaelis-Menten reactions functioning in the linear regime, the range of the V_{max} and K_m values can be extended with no change in the objective function, if both V_{max} and K_m are multiplied by the same positive constant. We have therefore considered a *correlated, multiplicative* perturbation scheme in which the parameters of the same reaction are multiplied by the same factor, namely $V_{maxi} = V_{max0i} \exp((2U_i - 1)a)$ $K_{mi} = K_{m0i} \exp((2U_i - 1)a)$, where V_{max0i}, K_{m0i} are the unperturbed parameters and U_i are independent, uniformly distributed in the interval $[0, 1]$.

For the data hereby presented, we have selected 10 sets of parameters corresponding to the 10 lowest values of the objective function. The corresponding unperturbed values of the parameters are represented as lines in the Figure S1. We have applied random perturbations (1000 samples for each parameter and for each parameter set) and chose an ϵ value such that the perturbed objective function is not larger than the largest objective function in the unperturbed set. Then, we represented the minimum and maximum perturbed values of the parameters as error bars superimposed on the unperturbed values profiles. The resulting plot gives an idea of the parameter uncertainty. Some confidence intervals are large, as expected. The confidence intervals transcribed in the Table 2 of the main text, are much larger, as they correspond to the minimum and maximum values of the perturbed parameters, all parameter sets confounded. For Table 2, we have chosen the correlated multiplicative perturbation scheme leading to the largest confidence ranges. Of course, this does not mean that choosing a parameter value at random in the ranges provided will provide a good objective function, because the represented confidence intervals are the projections onto the parameters directions of a complicated domain in the parameter space.

Figure S1

Dynamic ranges of substrate concentrations. The interval of substrate concentrations are presented in red and the magenta dots represent the positions of K_m. The

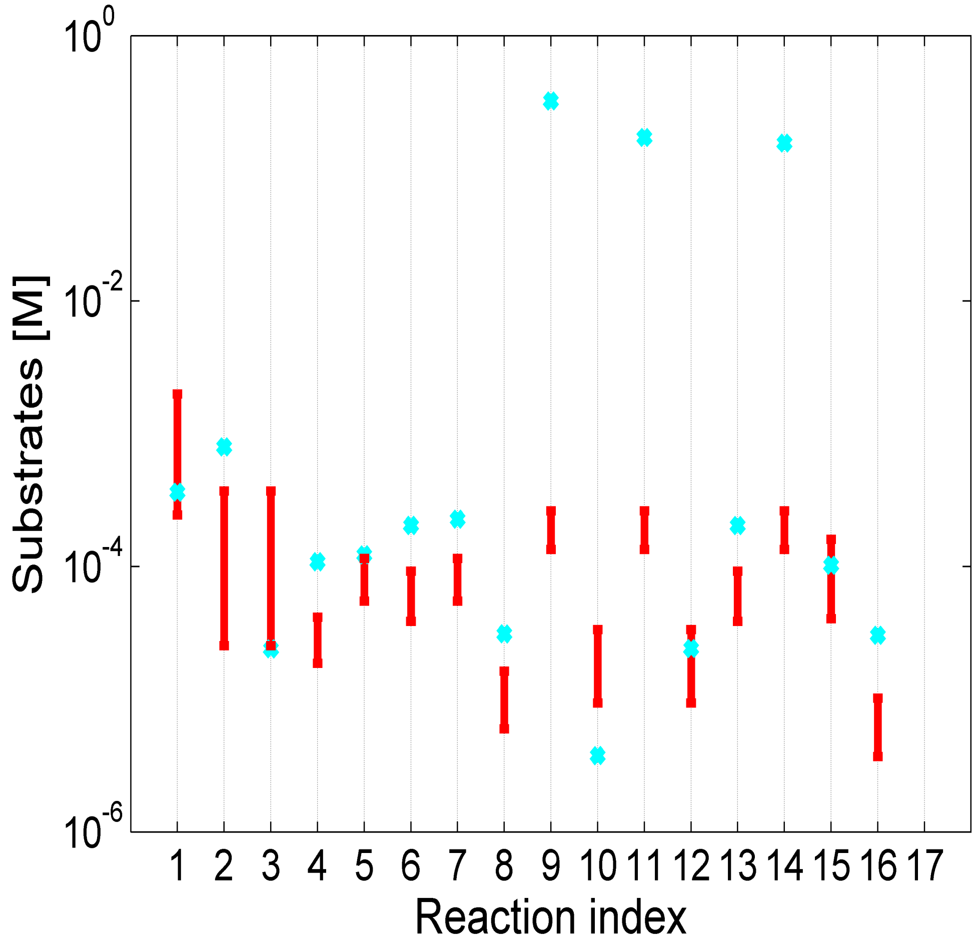

reaction 17 is not of Michaelis-Menten type and has not been represented in this plot. The dynamical concentration ranges could be supplemented by the zero concentrations (when it is supposed that the reactions rates are zero) and extended to $-\infty$ in logarithmic scale, but in this plot we have only considered non-vanishing concentrations, corresponding to effective measurements.

Figure S2

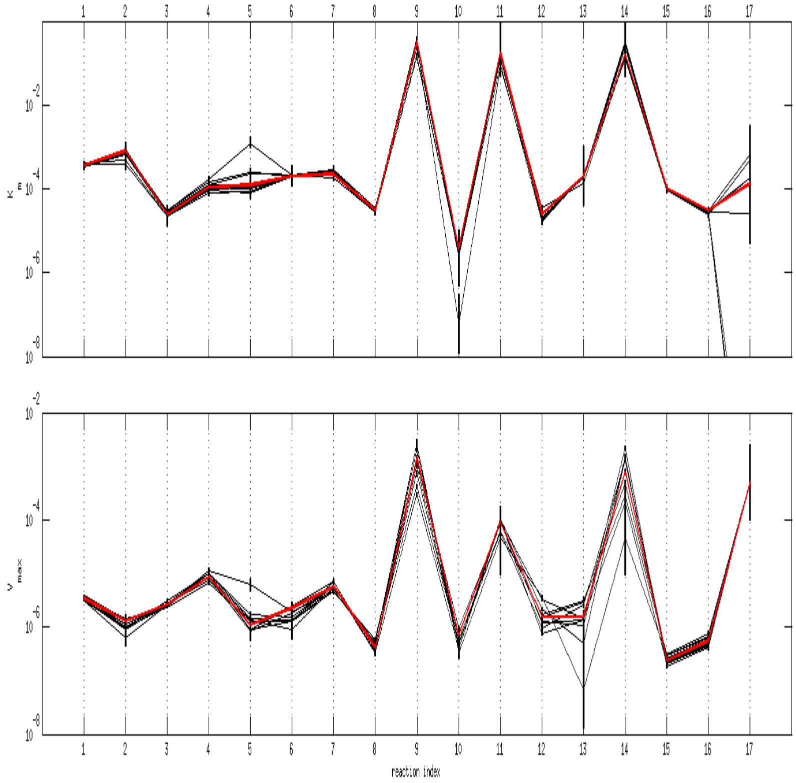

Parameter profiles and confidence intervals for a uncorrelated, multiplicative perturbation scheme. The lines connect parameter values corresponding to local minima of the objective function and therefore inform on the parameter correlation.

Figure S3

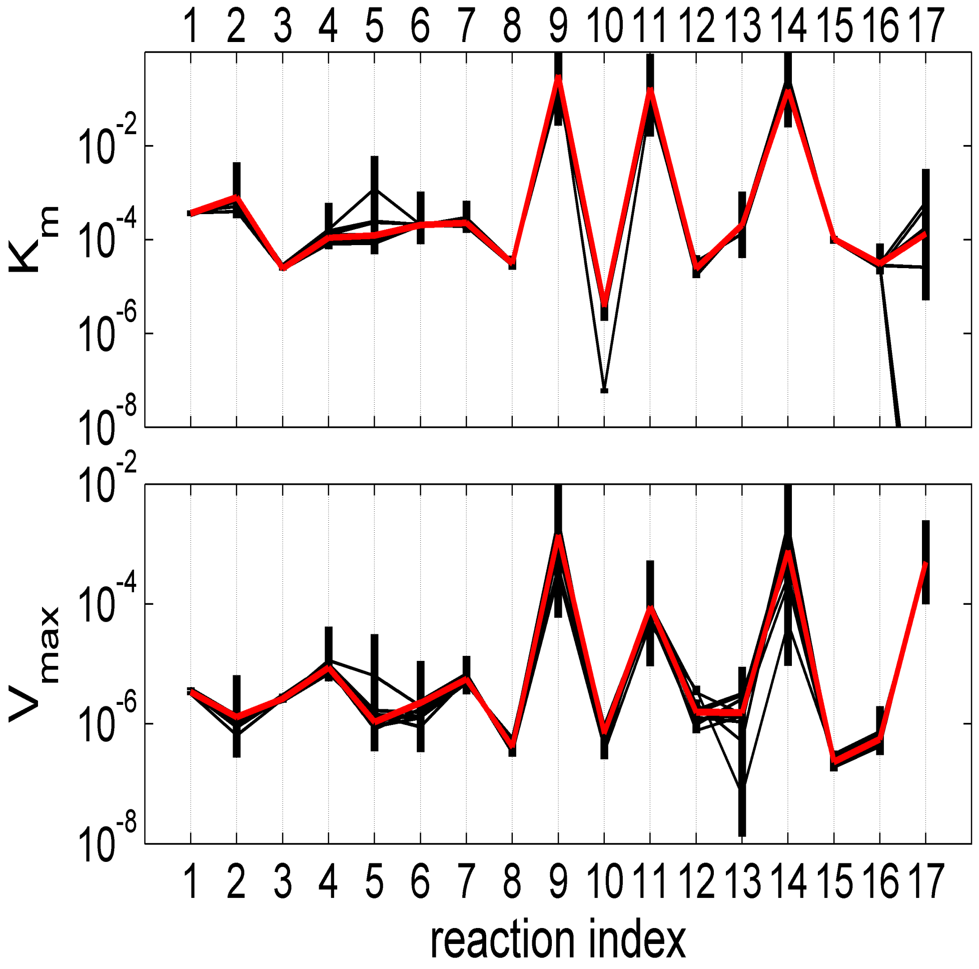

Parameter profiles and confidence intervals for a correlated, multiplicative perturbation scheme. Notice the larger confidence intervals

Table.S1

Flux sensitivity vs. V_{max}

	V_{max1}	V_{max2}	V_{max3}	V_{max4}	V_{max5}	V_{max6}	V_{max7}	V_{max8}	V_{max9}	V_{max10}	V_{max11}	V_{max12}	V_{max13}	V_{max14}	V_{max15}	V_{max16}	V_{max17}
R_1	0	0	0	0	0	0	0	0	0	0	0	0	0	0	0	0	0
R_2	0	0	0	0	0	0	0	0	0	0	0	0	0	0	0	0	0
R_3	0	0	0	0	0	0	0	0	0	0	0	0	0	0	0	0	0
R_4	0	0	0	0	0	0	0	0	0	0	0	0	0	0	0	0	0
R_5	0	0	0	0	0	0	0	0	0	0	0	0	0	0	0	0	0
R_6	0	0	0	0	0	0.58	0	0.44	0.05	0.37	0.02	-0.33	-0.38	-0.015	0.77	0.39	0
R_7	0	0	0	0	0	0	0	0	0	0	0	0	0	0	0	0	0
R_8	0	0	0	0	0	0	0	0.40	0	0	0	0	0	0	0.78	0.34	0
R_9	0	0	0	0	0	0.61	0	0.50	0.75	0.37	-0.03	-0.32	-0.34	-0.33	0.78	0.44	0
R_{10}	0	0	0	0	0	0.04	0	0.41	0.06	0.37	-0.002	-0.34	-0.02	-0.02	0.76	0.35	0
R_{11}	0	0	0	0	0	0.61	0	0.50	-0.25	0.37	0.96	-0.32	-0.34	-0.33	0.78	0.44	0
R_{12}	0	0	0	0	0	0.04	0	0.42	0.07	-0.66	-0.002	0.65	-0.02	-0.02	0.82	0.36	0
R_{13}	0	0	0	0	0	-0.42	0	0.44	0.05	0.37	0.02	-0.33	0.62	-0.01	0.77	0.39	0
R_{14}	0	0	0	0	0	0.61	0	0.50	-0.25	0.37	-0.03	-0.32	-0.34	0.67	0.78	0.44	0
R_{15}	0	0	0	0	0	0	0	0	0	0	0	0	0	0	1	0	0
R_{16}	0	0	0	0	0	0	0	0	0	0	0	0	0	0	0.88	0.30	0
R_{17}	0	0	0	0	0	0	0	0	0	0	0	0	0	0	0	0	0

Table.S2

Flux sensitivity vs. K_m

	K_{m1}	K_{m2}	K_{m3}	K_{m4}	K_{m5}	K_{m6}	K_{m7}	K_{m8}	K_{m9}	K_{m10}	K_{m11}	K_{m12}	K_{m13}	K_{m14}	K_{m15}	K_{m16}	K_{m17}
R_1	0	0	0	0	0	0	0	0	0	0	0	0	0	0	0	0	0
R_2	0	0	0	0	0	0	0	0	0	0	0	0	0	0	0	0	0
R_3	0	0	0	0	0	0	0	0	0	0	0	0	0	0	0	0	0
R_4	0	0	0	0	0	0	0	0	0	0	0	0	0	0	0	0	0
R_5	0	0	0	0	0	0	0	0	0	0	0	0	0	0	0	0	0
R_6	0	0	0	0	0	-0.57	0	-0.40	-0.05	-0.34	-0.02	0.33	0.38	0.01	-0.55	-0.33	0
R_7	0	0	0	0	0	0	0	0	0	0	0	0	0	0	0	0	0
R_8	0	0	0	0	0	0	0	-0.34	0	0	0	0	0	0	-0.57	-0.28	0
R_9	0	0	0	0	0	-0.61	0	-0.46	-0.75	-0.35	0.03	0.31	0.34	0.33	-0.56	-0.40	0
R_{10}	0	0	0	0	0	-0.04	0	-0.35	-0.06	-0.34	0.002	0.33	0.02	0.02	-0.56	-0.30	0
R_{11}	0	0	0	0	0	-0.61	0	-0.46	0.25	-0.35	-0.96	0.32	0.34	0.33	-0.56	-0.39	0
R_{12}	0	0	0	0	0	-0.04	0	-0.36	-0.07	0.63	0.002	-0.64	0.02	0.02	-0.59	-0.31	0
R_{13}	0	0	0	0	0	0.42	0	-0.40	-0.05	-0.34	-0.02	0.33	-0.61	0.01	-0.56	-0.34	0
R_{14}	0	0	0	0	0	-0.61	0	-0.46	0.25	-0.35	0.03	0.32	0.34	-0.67	-0.56	-0.40	0
R_{15}	0	0	0	0	0	0	0	0	0	0	0	0	0	0	-0.62	0	0
R_{16}	0	0	0	0	0	0	0	0	0	0	0	0	0	0	-0.61	-0.23	0
R_{17}	0	0	0	0	0	0	0	0	0	0	0	0	0	0	0	0	0

Table.S3

Concentration sensitivity vs. V_{max}

	$V_{max}1$	$V_{max}2$	$V_{max}3$	$V_{max}4$	$V_{max}5$	$V_{max}6$	$V_{max}7$	$V_{max}8$	$V_{max}9$	$V_{max}10$	$V_{max}11$	$V_{max}12$	$V_{max}13$	$V_{max}14$	$V_{max}15$	$V_{max}16$	$V_{max}17$
Ser	0	0	0	0	0	0	0	0	0	0	0	0	0	0	0	0	0
PS	0	0	0	0	0	-0.33	0	0.59	0.02	0.37	0.006	-0.31	-0.27	-0.002	0.82	0.53	0
Etn	0	0	0	0	0	0	0	0	0	0	0	0	0	0	0	0	0
PEtn	0	0	0	0	0	0	0	0	0	0	0	0	0	0	0	0	0
PE	0	0	0	0	0	0.71	0	0.64	-0.16	0.39	-0.02	-0.30	-0.23	-0.20	0.84	0.59	0
PC	0	0	0	0	0	0.02	0	0.55	0.03	-0.65	-6e-4	-0.33	-0.005	-0.004	0.83	0.49	0
Cho	0	0	0	0	0	0	0	0	0	0	0	0	0	0	1.1	-0.65	0
PCho	0	0	0	0	0	0	0	-0.50	0	0	0	0	0	0	0.91	0.49	0

Table.S4

Concentration sensitivity vs. k_m

	K_{m1}	K_{m2}	K_{m3}	K_{m4}	K_{m5}	K_{m6}	K_{m7}	K_{m8}	K_{m9}	K_{m10}	K_{m11}	K_{m12}	K_{m13}	K_{m14}	K_{m15}	K_{m16}	K_{m17}
**Ser**	0	0	0	0	0	0	0	0	0	0	0	0	0	0	0	0	0
**PS**	0	0	0	0	0	0.33	0	-0.57	-0.02	-0.36	-0.006	0.31	0.27	0.002	-0.57	-0.50	0
**Etn**	0	0	0	0	0	0	0	0	0	0	0	0	0	0	0	0	0
**PEtn**	0	0	0	0	0	0	0	0	0	0	0	0	0	0	0	0	0
**PE**	0	0	0	0	0	-0.71	0	-0.62	0.160	-0.38	0.02	0.30	0.23	0.20	-0.58	-0.56	0
**PC**	0	0	0	0	0	-0.02	0	-0.52	-0.032	0.64	6e4	0.32	0.005	0.004	-0.58	-0.45	0
**Cho**	0	0	0	0	0	0	0	0	0	0	0	0	0	0	-0.66	0.62	0
**PCho**	0	0	0	0	0	0	0	0.47	0	0	0	0	0	0	-0.61	-0.46	0
